# Peptide Nanovesicles Formed by the Self-Assembly of Branched Amphiphilic Peptides

**DOI:** 10.1371/journal.pone.0045374

**Published:** 2012-09-18

**Authors:** Sushanth Gudlur, Pinakin Sukthankar, Jian Gao, L. Adriana Avila, Yasuaki Hiromasa, Jianhan Chen, Takeo Iwamoto, John M. Tomich

**Affiliations:** 1 Department of Biochemistry, Kansas State University, Manhattan, Kansas, United States of America; 2 Division of Biochemistry, Core Research Facilities, Jikei University School of Medicine, Tokyo, Japan; Consejo Superior de Investigaciones Cientificas, Spain

## Abstract

Peptide-based packaging systems show great potential as safer drug delivery systems. They overcome problems associated with lipid-based or viral delivery systems, *vis-a-vis* stability, specificity, inflammation, antigenicity, and tune-ability. Here, we describe a set of 15 & 23-residue branched, amphiphilic peptides that mimic phosphoglycerides in molecular architecture. These peptides undergo supramolecular self-assembly and form solvent-filled, bilayer delimited spheres with 50–200 nm diameters as confirmed by TEM, STEM and DLS. Whereas weak hydrophobic forces drive and sustain lipid bilayer assemblies, these all-peptide structures are stabilized potentially by both hydrophobic interactions and hydrogen bonds and remain intact at low micromolar concentrations and higher temperatures. A linear peptide lacking the branch point showed no self-assembly properties. We have observed that these peptide vesicles can trap fluorescent dye molecules within their interior and are taken up by N/N 1003A rabbit lens epithelial cells grown in culture. These assemblies are thus potential drug delivery systems that can overcome some of the key limitations of the current packaging systems.

## Introduction

Lipid-based carriers such as liposomes and micelles have traditionally been the preferred methods of choice for delivering bioactive compounds into living systems [1]. However, despite certain advantages, lipid-based vesicles possess a number of shortcomings, especially with regard to stability, bioreactivity and toxicity [2]. Cationic liposomes especially, are known to trigger specific signaling pathways, which involve PKC [3], stimulate TLR4 in dendritic cells [4] or directly bind to membrane lipids and modulate the activity of membrane proteins [5], [6]. Virus mediated delivery of genetic material has made much progress but still suffers from many serious drawbacks, which include immunogenicity [7]–[9], lack of specificity [10] and insertional mutagenesis [11] leading to such adverse effects as the development of certain forms of cancer.

Recent research on self-assembling polymeric vesicles show promise in replacing liposomes and other lipid-based delivery systems as a means for targeting cells or tissues [12]. Self-assembling polymeric vesicles have displayed improved stability, specificity and tune-ability over lipid-based vesicles [13] and are seen as attractive candidates for drug delivery. Polymeric vesicles can be obtained from a variety of self-assembling molecules [12]–[17] of which, vesicles assembled from amphiphilic block copolymers are the most widely studied. Amphiphilic block copolymers are high molecular weight, linear hybrids with distinct hydrophobic and hydrophilic segments. The amphiphilic blocks of these block copolymers can be synthetic or non-synthetic. When a polypeptide is used as one of the segments, the self-assembled vesicular structures are termed peptide vesicles or peptosomes. In most cases, the polypeptide segment is linked to a synthetic hydrophobic segment, which drives the assembly. While block copolymer monomers composed entirely of polypeptides, like Ac-V_m_K_n_-NH_2_, Ac-G_m_D_n_-OH, Ac-V_6_D-OH, Ac-KA_6_-OH have been reported to form vesicles, these examples are only a few when compared to the many synthetic polymers that are capable of self-assembling into vesicles. Vauthey *et al.* (2002) [18] were the first to show that a simple 7–8 residue amphiphilic peptide is capable of self-assembling into nanotubes and nanovesicles. They called these peptides, “Surfactant-Like Peptide”. Santos *et al*. (2002) [19], designed similar peptides as the ones by Vauthey *et al.* (2002) with glycine and aspartic acid that were capable of self-assembling into nanotubes and nanovesicles. van Hell *et al*. (2007) [20], showed the self-assembly of an amphiphilic oligopeptide SA2 into a nano-sized vesicle. These vesicles not only carry the advantage of being more biocompatible and biodegradable than other synthetic and semi-synthetic polymer vesicles, but are more stable than lipid and polysaccharide vesicles. Excellent reviews on polymeric vesicles and self-assembly of peptide amphiphiles discuss the various facets of such supramolecular assembly [12], [13], [21]–[23].

The sequences presented here add a new peptide motif to the existing field of peptide vesicles. They are a set of self-assembling branched molecules composed entirely of naturally occurring amino acids. They contain a cationic head attached to branched hydrophobic segments thus mimicking bilayer-forming phosphoglycerides in overall architecture. The relatively short sequences form extremely stable β-like complexes, and are easy to modify chemically. As self-assembling molecules composed entirely of amino acids they have the advantage of being more biocompatible than other non-natural polymeric based vesicles. While it is more common for self-assembling molecules to be designed to adopt an α-helical conformation during assembly, a few β-structure stabilized spherical assemblies have been reported and studied. They are a relatively new area of research and there are few examples in the literature describing β-structure forming peptides or proteins adopting a vesicular morphology (e.g. silk micro- [24] and nano-spheres [25], spherulites [26] and amylospheroids [27]). In silk micro & nano-spheres, the β-sheet cross-linking helps form stable structures for delivering drugs and other small molecules.

**Figure 1:**
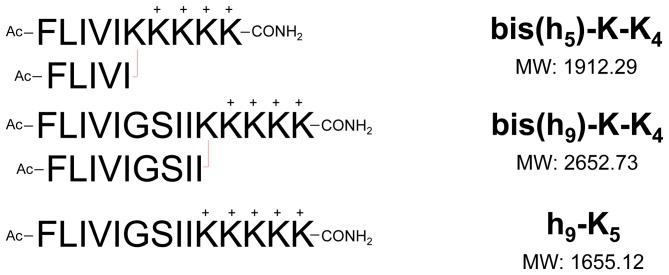
Assembling and control sequences. The peptide assemblies are an equimolar mixture of the acetylated bis(h_9_)-K-K_4_ and bis(h_5_)-K-K_4_ sequences unless otherwise stated. The linear h_9_-K_5_ control sequence does not associate to give any type of structure.

Our peptides (Fig. 1) adopt an apparent vesicular morphology, stabilized by β-like structure. The peptide sequences used for these self-associating assembles evolved from earlier studies on several adhesive peptides [28], [29]. Previously, we identified several peptide constructs that produced nano-fibrils with mechanical adhesive properties due to entanglement [30]. The hydrophobic core sequence used in the adhesives occurs in nature as an internal fragment of the human dihydropyridine sensitive L-type calcium channel segment, CaIVS3 (DPWNVFDFLIVIGSIIDVILSE). The underlined segment is a portion of a transmembrane helix that contributes to the central water-filled pore of this channel [31], [32]. Lyophilization of the CaIVS3 peptide resulted in insoluble clumps resistant to mechanical disruption. This property suggested that strong cohesive forces in the absence of water were driving this association. As linear non-branched sequences flanked by tri-lysyl segments (i.e. KKK(h_5_ or h_9_)KKK) these sequences proved to be strong β-structure formers in water at high pH, assembling into large β-sheet assemblies as judged by analytic ultracentrifugation studies [29].

In this study, the adhesive sequences were redesigned to be purely amphiphilic with an oligo cationic pentapeptide at the C-terminus containing a branch-point for the attachment of two hydrophobic adhesive sequences FLIVIGSII and FLIVI in a parallel array (Fig. 1).

## Results

### Design and Synthesis of Assembling and Control Peptides

The amphiphilic peptides employ an oligo-lysine (K = 5) segment with the α- and ε-amino groups of the N-terminal lysyl-residue acting as the branch point for the addition of either two nine- (FLIVIGSII) or five- (FLIVI) residue hydrophobic sequences. The Fmoc-based chemical synthesis utilized four lysine residues with ε-amine *t*-Boc protecting groups while the branching lysyl group contained Fmoc groups at both α- and ε-amine groups. The chemical release of the two Fmoc protecting groups on the lysine allowed for the simultaneous addition of identical amino acids to the now bifurcated chain, thereby permitting the synthesis of the bis-N-terminal hydrophobic tails. Several variants of the peptides were prepared, which included the N-terminal phenylalanines blocked by either acetylation or covalent attachment of a fluorescent dye and sequence variants. Data presented here were obtained using N-termini acetylated peptides unless otherwise noted. The stepwise addition of amino acids to both the α- and ε-amine of a lysine was previously described in Iwamoto et al. (1994) [32].

**Figure 2:**
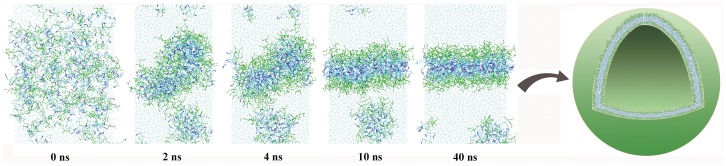
A coarse-grained simulation of bis(h_5_)-K-K_4_ bilayer self-assembly. All lysine residues are plotted in green ball-and-stick representations, the aromatic sidechains of phenylalanine residues in blue sticks, and all other residues in cyan. The snapshots were taken at 0 ns, 2 ns, 4 ns, 10 ns and 40 ns, respectively. A hypothetical peptide vesicle model is shown to the right.

Based on the similarity in molecular architecture of our peptides to phospholipids, we hypothesize that above certain concentrations, analogous to the critical micellar concentration (CMC) observed for phospholipids, the peptides undergo supramolecular self-assembly with the hydrophobic segments of these peptides initially driving the formation of ordered bilayer delimited, spherical water-filled structures. It is during the assembly process that solutes present in the water become encapsulated. The positively charged lysines face the two aqueous phases, and the hydrophobic residues stabilizing the structure as extended strands with inter- and intra-molecular hydrogen bonds. In addition to the weaker hydrophobic interactions that stabilize lipid bilayers, these assemblies appear to form parallel hydrogen-bonded networks between adjacent β-structures (see CD data) that help maintain the assemblies at much lower concentrations (low micromolar), conditions where most phospholipid assemblies would be fully dissociated. Course-grained molecular dynamic simulations support the comparison between these peptides and diacyl-phospholipids. Figure 2 summarizes a representative simulation of the self-assembly process for one of the peptide- bis(h_5_)-K-K_4_, which experimentally, leads to stable β-like assemblies in solution. Starting from a completely random distribution in water, the peptides rapidly begin to segregate and form a discernable bilayer structure within ∼40 ns. The equilibrium simulations of the assembled model bilayers suggest that these peptides have similar lateral dimensions compared to common phospholipids. Examining the modeled structure revealed no inter-digitation between opposing side-chains. Since these are course-grained simulations no inferences can be drawn as to hydrogen bonding patterns.

### Preparation and Characterization of Peptide Assemblies

Preparation of the peptide vesicles is done in a fashion similar to that of liposome preparation. It commenced with the mixing of the two peptides, bis(h_9_)-K-K_4_ and bis (h_5_)-K-K_4_, individually dissolved in 40% 2,2,2, Trifluoroethanol (TFE). Under these conditions the peptides adopt a helical conformation, thereby forcing the peptides into a monomeric state, thus ensuring proper mixing.

The peptide assemblies can be prepared from the bis(h_9_)-K-K_4_ and bis(h_5_)-K-K_4_ peptides by themselves or in combination. Mixing of the longer and shorter peptide chains is analogous to the preparation of lipid bicelles [33]. We generally used an equimolar mixture of the two peptides (N-termini blocked) to reduce potential strain due to the high curvature found in nano-vesicles. We observed that these preparations also resulted in better samples for collecting the Transmission Electron Microscope (TEM) images.

Once combined, the TFE/peptide solution was briefly vortexed and allowed to stand for two hours before removing the solvent under vacuum. The dried samples were redissolved with the drop-wise addition of water. The rehydrated samples were vortexed briefly, adjusted to pH 7 with dilute NaOH solution and allowed to stand for at least two hours before using. Peptide concentrations were determined using the molar absorptivity (ε) of phenylalanyl residues in the sequence (195 cm^−1^ M^−1^ at 257.5 nm).

**Figure 3:**
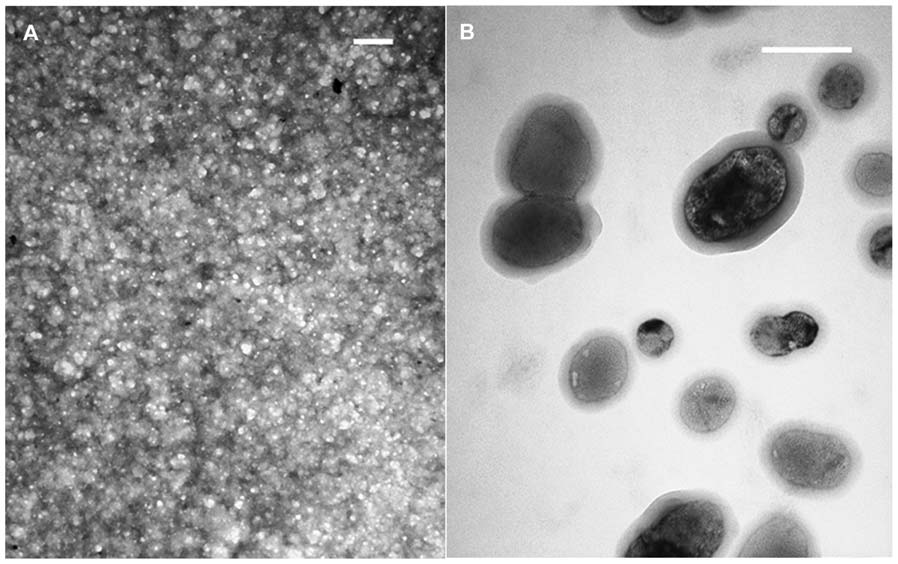
Transmission Electron Micrograph (TEM) of h_9_h_5_-vesicles. These peptides self-assemble into nano-sized vesicles. TEM images of (A) a concentrated and (B) a diluted sample of the peptide mixture, stained with 5% phosphotungstic acid and Osmium tetroxide (OsO4) vapors, respectively (200 nm scale bar).

From this point on, the equimolar ratio of the peptide mixture will be referred to as “h_9_h_5_-vesicles”. In Figure 3 panels (A–B), TEM images are shown for a concentrated (1.6 mM each) and diluted (25 µM each) preparation of h_9_h_5_-vesicles, respectively. The TEM images revealed that the h_9_h_5_-vesicles adopt a spherical morphology with a fairly uniform size distribution between 50–150 nm, a size on the order of adenovirus capsid particles (40–80 nm in diameter) [34]. The concentrated sample (Fig. 3A) appears as a densely packed layer that resembles fish roe. In Figure 3B, under dilute conditions, individual liposome-like structures are visible with some appearing to be fusing and others, which are larger and more oblong, as though they have completed the fusing process. This property of membrane fusion is more common to lipid vesicles and appears to be possible with the peptide assemblies, thereby providing an indication that the h_9_h_5_-vesicles are forming a membrane-like structure with a water filled interior.

**Figure 4:**
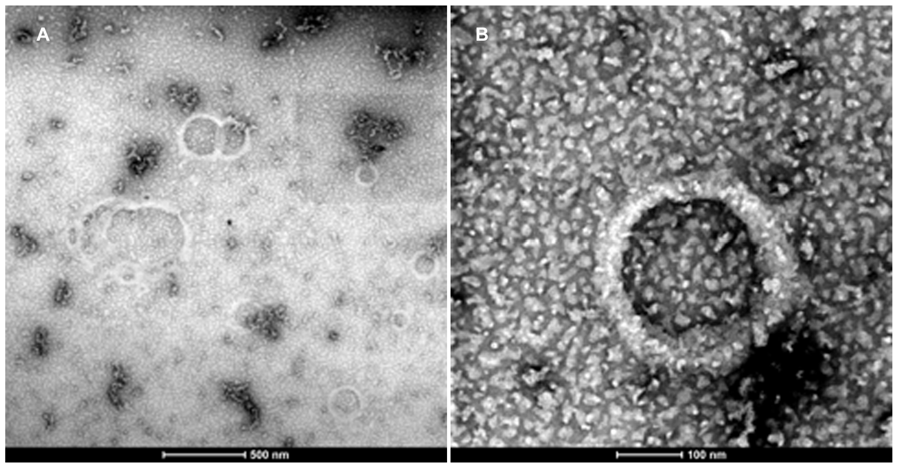
Scanning Transmission Electron Micrograph (STEM). Vesicles were prepared with 30% CH_3_-Hg label in both the h_5_ and h_9_ peptides at 0.1 mM concentration were negatively stained using a multi isotope 2% Uranyl acetate (Uranium bis(acetato)-O)dioxo-dihydrate) aqueous solution. The images were captured using annular dark field mode was then inverted to produce the final image.

Figure 4A shows the scanning transmission electron micrograph (STEM) of a number of vesicles labeled with methyl mercury (CH_3_-Hg). The vesicles contain unlabeled (70%) and Hg-labeled (30%) 1∶1 h_5_:h_9_ peptides. The CH_3_-Hg labeled vesicles (∼100–500 nm diameter) are clearly visible on the amorphous carbon substrate carbon grid surface. A number of individual vesicles are seen as well as two larger associations. In Figure 4B, a higher resolution STEM of a single vesicle is shown. This roughly 200 nm diameter hollow peptide assembly shows the Hg-label only at the outer edge of the vesicle. The interior is free of peptide. Since both peptides are labeled, the CH_3_-Hg should be present on both sides of the bilayer, unfortunately at this level of resolution the electron densities associated with the labeling atom cannot be resolved. Energy-dispersive X-ray spectra (EDX) were obtained for all samples (data not shown) and showed the presence of mercury along with copper from the grid and uranium from the uranyl acetate.

**Figure 5:**
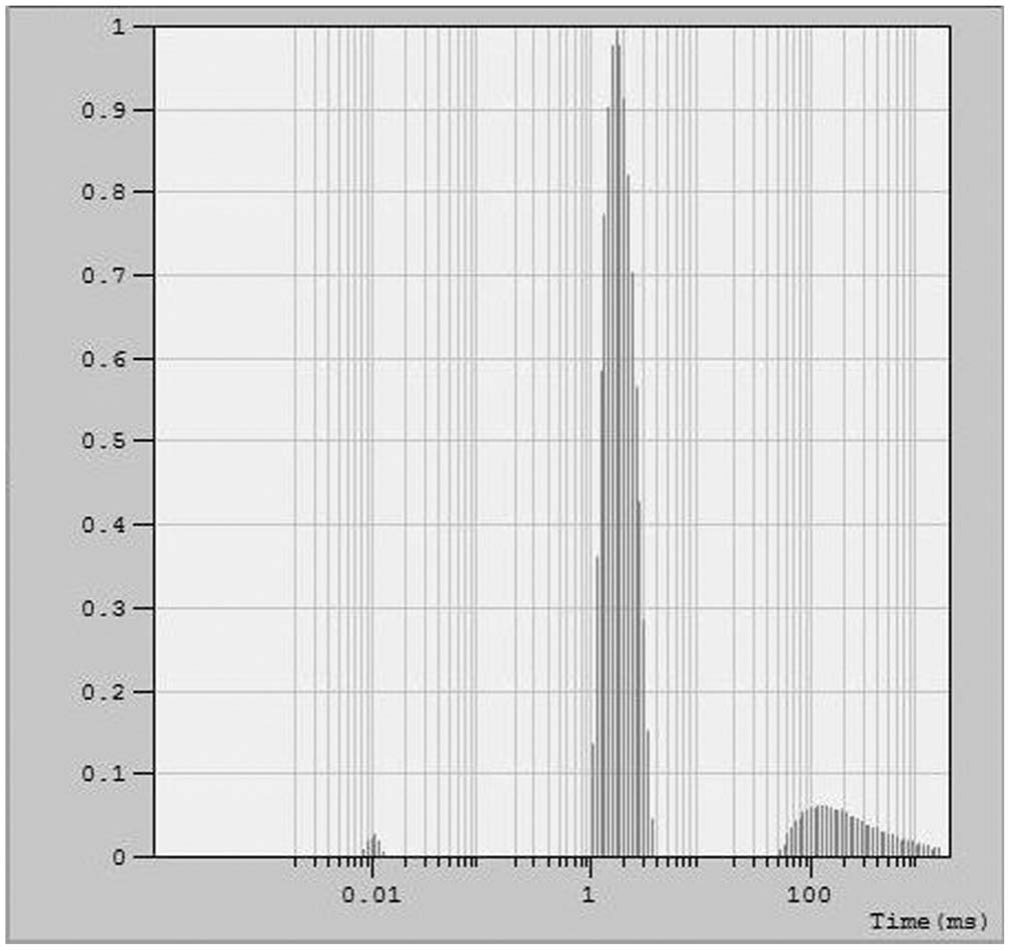
Dynamic Light Scattering of h_9_h_5_-vesicles. Distribution analysis of the data showing two major distributions centered around 1.68 ms and 119.4 ms and a mean correlation time of 1.85 ms and 275.7 ms respectively. These correlation times correspond to a hydrodynamic radius of 80 nm and 10 µm respectively.

The size distribution observed in the EM images is in agreement with dynamic light scattering (DLS) data, obtained using identically prepared samples. DLS data for a concentrated sample of the peptide vesicles yielded an average hydrodynamic radius of 80 nm. The hydrodynamic radius of the sample was obtained by performing curve fitting and analyzing the distribution, using Dynals (see Methods) yielded a mean auto correlation time of 1.85 ms for the major distribution (Fig. 5).

**Figure 6:**
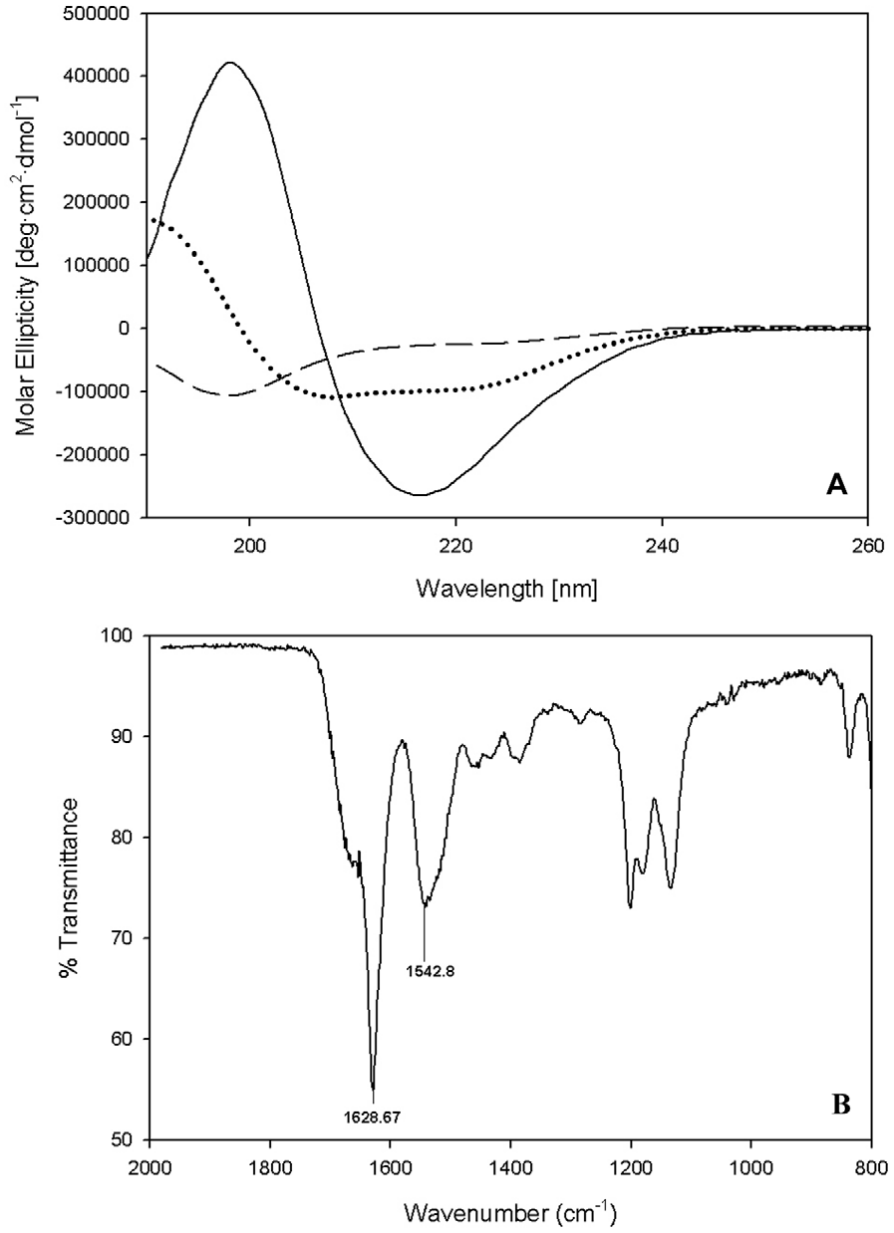
Secondary Structural Characterizations. (A) Circular dichroism (CD) spectroscopy shows that the h_9_h_5_-vesicles adopt a β-structure (solid line) at pH 7.0 in 5 mM NaHCO_3_, a helical structure (dotted line) in 40% TFE and remains unstructured (dashed line) when lacking the lysine branch point (h_9_-K_5_). (B) FTIR spectroscopy of dried material shows the characteristic 1629 cm^−1^ maximum corresponding to β- secondary structure.

Circular dichroism (CD) spectra support the β- or extended conformation of the assembled branched-peptides structures (Fig. 6A, solid line) at pH 7 in 5 mM NaCO_3_ solution, with a bisignate profile characteristic of a β-structure with a minimum at 218 nm and maximum at 198 nm. However, when dissolved in 40% TFE, the peptides adopt helical-like conformations with the characteristic α-helix minima at 208 nm, 222 nm, and a maximum at 190 nm (Fig. 6A, dotted line). The linear sequence (h_9_-K_5_), lacking the branching point, appears to have a random structure (Fig. 6A, dashed line) at pH 7 in 5 mM NaCO_3_ solution. It failed to show any type of assembly in TEM images or assembly in the analytic ultracentrifuge.

To estimate the secondary structure content, CD spectra were analyzed using a constrained least squares fitting analysis program, LINCOMB [35], which uses polypeptide based reference sets. The analysis showed the linear peptide to have 32.5% random and 12% sheet component with the remaining assigned to turn(I). The assembled branched-peptides structures in 5 mM NaHCO_3_ on the other hand turned out to have a sheet component close to 93% with the remaining assigned to turn(I). In 40% TFE, the assembled branched-peptides structures showed a high helix component of 63% with 30% random and the remaining assigned to turn(I).

These data support the requirement of branched sequences for assembly and that these sequences are able to undergo reversible transitions from helix (monomer) to beta structure (assemblies) depending on solvent conditions. Based on the data presented here, it is not very clear how the branching helps in supramolecular assembly but we believe chain entanglement, the 90° offset of the branch points and hydrophobicity play important roles in the three dimensional assembly. The h_9_h_5_-vesicles are very stable, resisting the action of SDS, urea and trypsin, as determined by the absence of change in the CD spectra before and after treatment. Once assembled, the h_9_h_5_-vesicles can be switched from a pure aqueous solution to one containing 40% TFE or ethanol thereby yielding a CD spectrum with the hallmark α-helical minima and maximum, indicating a conversion between β- and α-structures thereby giving us the ability to release and quantify the contents of entrapped molecules.

ATR-FTIR was used to further confirm the peptide secondary structure in the assembled state. The spectrum of the dry powder of the peptide mixture (Fig. 6B) revealed a single amide-I stretch at 1629 cm^−1^. Normally, antiparallel β-structures possess a principle β-sheet amide-I stretching vibration maximum at 1630–1640 cm^−1^ [36], [37] with a secondary absorption at a frequency 50–70 cm^−1^ higher. The absence of the secondary absorption in Figure 6B is consistent with the branched peptides adopting a parallel β-structure orientation. This result is consistent with those observed from the self-assembly simulations (Fig. 2). These results also indicate that the dry peptide mixture retains its secondary structure in the absence of bulk solvent, an important property when long-term storage and ease of transport of encapsulated materials is under consideration. The amide-I band component was analyzed for the estimation of secondary structure content. Curve fitting of the second derivative spectra using OriginPro 8.6 (Nothampton, MA) revealed that the dry powder of the peptide mixture contained about 47% β-sheet, calculated by the relative area at 1629 cm^−1^ and the remaining assigned to beta turn, calculated by adding all the β-turn bands between 1660 and 1690 cm^−1^.

**Figure 7:**
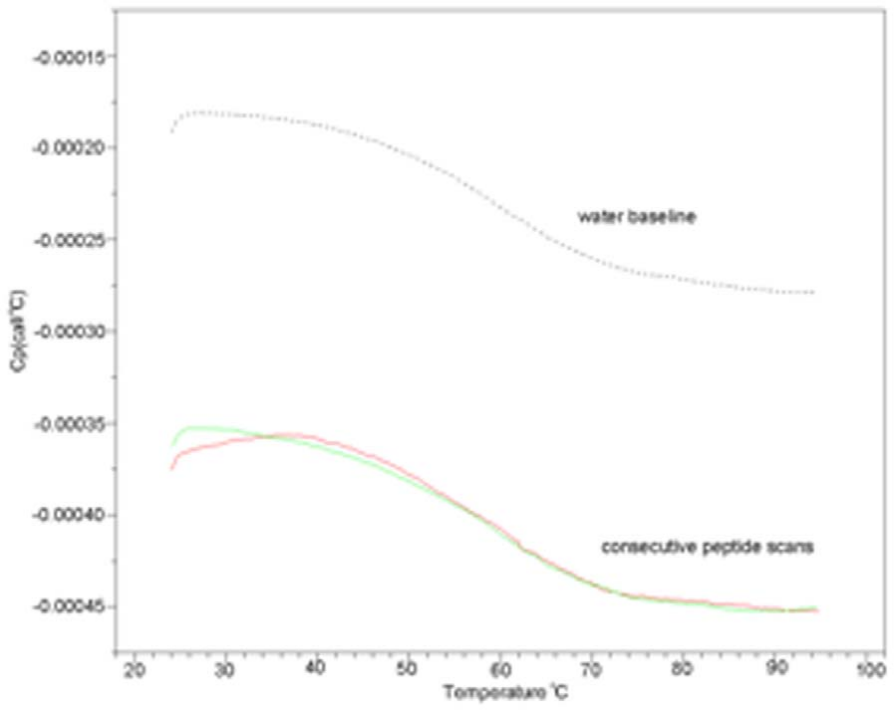
Differential scanning calorimetry of 1.0 mg/ml peptide in water. Heat capacity of solution is shown as a function of temperature. The instrument water baseline is shown together with the thermograms obtained during two consecutive upward temperature scans.

Temperature dependence on the unfolding of the peptides were determined by Differential Scanning Calorimetry (DSC) and showed no visible transitions in the temperature range 20–95°C (Fig. 7). The peptides displayed the expected heat capacity lower than water. However, the shape of the three temperature scans were exactly as that observed with just water. The peptide concentrations were kept high (1.0 mM) to eliminate any issues related to sensitivity. The lack of any temperature transition indicates that no cooperative change in structure occurred over the temperatures tested. Lipids in liposomes show such transitions in the 35–45°C [38]. At these melting temperatures, lipid vesicles tend to get leaky and such a property can be seen as disadvantageous for its inability to retain its contents at higher temperatures. The h_9_h_5_-vesicles on the other hand appear stable over a wide range of temperatures and this property can be advantageous when being used as a delivery system *in vivo*.

### Encapsulation of 5(6)-Carboxyfluorescein

**Figure 8:**
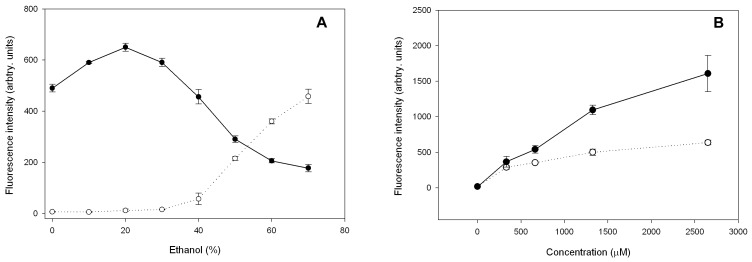
Encapsulation studies with 5(6)-Carboxyfluorescein. (A) 5(6)-Carboxyfluorescein loaded peptide vesicles were prepared in different ethanol concentrations and separated over 30 kDa MWCO filters. Fluorescence intensities of the filtrate (open circles) and retentate (solid circles) were collected after separation and show that encapsulation efficiency decreases with increasing ethanol concentration. (B) h_9_h_5_-vesicles prepared with the dye present during vesicle formation (solid circles) or added after the vesicles are formed (open circles). Each data point represents an average of 3 separate experiments performed on different days. Data points are connected with straight lines through their midpoints.

We investigated the ability of h_9_h_5_-vesicles to encapsulate small molecules by preparing them first in the helix inducing solvent, ethanol, and then diluting them with water to lower the organic solvent concentration to a level that permits β-structure formation thereby simultaneously encapsulating the dye 5(6)-Carboxyfluorescein. Dried peptide mixture and the dye were initially dissolved in 100% ethanol and eventually diluted with water to achieve appropriate final solvent concentrations ranging from 70% - 10%. Any non- encapsulated or surface-bound dye was removed by centrifuging the sample through a 30 pre-wetted kDa molecular weight cut off (MWCO) filter. The encapsulation efficiency, as followed by the fluorescence intensities of the encapsulated dye, decreased with higher ethanol concentrations (Fig. 8A) and was due to the inability of the peptides to self-assemble into vesicles at such ethanol concentrations. CD spectra confirmed the secondary structure transition from predominantly helical at high ethanol concentrations to more beta or extended structure upon dilution. To avoid fluorescence quenching effects due to locally high concentrations of the dye within the vesicles, the fluorescence intensities of the final samples were collected in pure TFE where there are no supramolecular assemblies.

In a separate experiment, to differentiate dye encapsulation from dye binding, 5(6)-Carboxyfluorescein was added at two different stages of vesicle formation. The first one involved preparing the h_9_h_5_-vesicles in the presence of the dye and in the second, the dye was added after the vesicles were formed. The rationale involved saturating the binding sites on the outside of the h_9_h_5_-vesicles with increasing dye concentration. Binding increases until at a certain dye concentration the surface is saturated and no more dye binds. On the other hand, if the dye is encapsulated during vesicle formation, it will continue to show an increase in dye intensity until the dye reaches its solubility limit. Figure 8B shows the results of increasing concentrations of the dye, 5(6)-Carboxyfluorescein, encapsulated into and bound to h_9_h_5_-vesicles that were prepared by different methods described above. The results indicate that considerably more dye is incorporated under conditions where both encapsulation and surface binding occur (Fig. 8B, solid circles) as compared to when the dye added to preformed vesicles (Fig. 8B, open circles). The concentration of the peptide in the two samples remained constant indicating that fluorescence intensity changes of the dye were due to dye binding and encapsulation and not due to peptide-dye interaction. This result clearly indicates that the peptide vesicles are capable of encapsulating small water-soluble dye molecules within their water-filled interior.

### Peptide Assemblies as Delivery Vehicles *in vitro*

**Figure 9:**
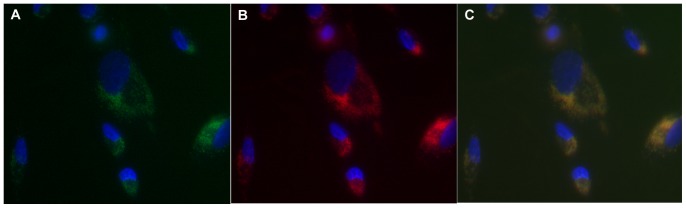
Cellular uptake of preloaded h_9_h_5_-vesicles. (A–C) h_9_h_5_-vesicles with one of the peptides labeled (bis(h_5_)-K-K_4_ labeled with Carboxytetramethylrhodamine) and loaded with 5(6)-Carboxyfluorescein were delivered to N/N 1003A rabbit lens epithelial cells. Green and red filter settings were used for the detection of 5(6)-Carboxyfluorescein (A) and Rhodamine labeled peptide (B) respectively. (C) is a merge of a & b showing co-localization of dye and h_9_h_5_-vesicles within the cell.

The potential use of h_9_h_5_-vesicles as a delivery system was explored, *in vitro* with preloaded assemblies containing fluorescent compounds. The loading efficiency was measured with three different fluorescent compounds, 5(6)-Carboxyfluorescein, tryptophan and Rhodamine 6G. Non-encapsulated or bound compounds were removed by gel filtration. The calculated trapping efficiency was approximately 5 to 8% with the generation of 10^10^ to 10^11^ assemblies, using a 1.6 mM concentration of h_9_h_5_-vesicles. Vesicular uptake was followed using N/N 1003A rabbit lens epithelial cells (Fig. 9A–C) grown on cover slips (in twelve well cell culture plates). Control cells, incubated with either free dye or dye mixed with the linear peptide (h_9_-K_5_) showed no incorporated fluorescence (image not shown). In Figure 9A–C, h_9_h_5_-vesicles containing encapsulated fluorescein were prepared with a portion (50%) of the (bis(h_5_)-K-K_4_) peptide’s N-termini covalently attached to the fluorescent dye- Carboxytetramethylrhodamine. These vesicles were then incubated with N/N 1003A lens epithelial cells. This experiment was designed to look for the co-localization of the trapped soluble dye with the peptide assemblies labeled with a different fluorescent dye. The images shown are of the same cells visualized with different filter settings to check for co-localization of the two dyes. After a 10 h incubation period, encapsulated 5(6)-Carboxyfluorescein (Fig. 9A) was made visible when viewed with a green filter indicating that the cells had internalized the dye. In Figure 9B the cells were imaged using a red filter also showing the cellular uptake of the covalently labeled peptide assemblies. Figure 9C shows the color merge of 9A & 9B, indicating co-localization of the soluble dye and the rhodamine-labeled peptide within the cell. Co-localization indicates that the intact loaded peptide vesicles enter the cytoplasm rather than releasing their contents through a fusion-like mechanism at the plasma membrane. Due to the limited exposure time, these experiments gave no indication that the dye was released from the vesicles within the cell. All we can demonstrate with this initial line of investigation is that the soluble dye has been delivered to the interior of the cell.

The final peptide concentration used in these experiments was 50 µM. There was no apparent toxic effect on the cells at these concentrations. Cell cytotoxicity assays were carried out with increasing concentrations of the peptide (1 µM –1 mM), and showed no cell death up to 50 µM. Cell cytotoxicity assays were measured using the well-established trypan blue dye exclusion assay. While cellular uptake of lipid vesicles occurs via membrane fusion and the subsequent release of the vesicle contents directly into the cytoplasm, cellular uptake of the h_9_h_5_-vesicles described here occurs most likely via endocytosis. However, the addition of multiple addressable functional groups could allow for the attachment of receptor targeting molecules, such as biotin, thereby helping to target the vesicles to specific cell types.

## Discussion

To our knowledge, no other branching amphiphilic peptides have been successfully designed, simulated and tested that spontaneously form aqueous-filled vesicles. By combining an oligolysine tail with two strong β-forming hydrophobic sequences we have identified such an ensemble. The single-chained linear version of the peptides failed to show any lipid- or detergent-like behavior. One might have expected the linear sequence to form micelles, however this was not the case. Clearly, the branched-arrangement of the hydrophobic sequences is essential for assembly. The hydrophobic sequences used in this study had previously been found to undergo pH dependent aggregation and adhesive qualities when flanked by oligo-lysine segments (i.e. KKKFLIVIKKK) [29]. Adding two of these sequences to a lysine residue generated a branched sequence that mimicked certain properties of phospholipids: the ability to form bilayer –like structures and under certain conditions, and fuse to increase in size. Also as the peptides transitioned from monomer to oligomer (helix to beta-like structure) during dilution of the ethanol, the oligomerizing peptides surround and entrap nearby solutes in a manner similar to that seen with lipid vesicles. Adding alcohols back to the vesicle containing solution reversed the process thereby releasing the contents of the assemblies.

Our conclusions regarding the assembled structure are based on the amphiphilic diacyl lipid-like design of the peptides, computer simulations, stability of the assemblies, the fact that higher amounts of water-soluble dyes were trapped when incubated with assembling peptides and the subsequent release of trapped material under conditions where the peptides transitioned from assembled β back to monomeric α structure at higher ethanol concentrations. While the cationic nature of the assemblies facilitates satuarable surface binding of anionic dyes, the amount of dye trapped in this process far exceeds that of the dye amount bound to preformed vesicles. Cryo-EM was not successful in visualizing hydrated samples and freeze etching was unsuccessful in splitting the proposed bilayer structure. Labeling the two peptides with mercury and then analyzing 30% labeled samples using STEM has provided the best evidence to date that the peptides form an ordered structure that defines the boundary surrounding the vesicles. The amphipathic nature of the molecules would allow the peptides to form one of two potential structures–a micelle with all of the hydrophobic segments filling the structure and excluding water or a bilayer that would allow for the formation of a water-filled structure. The structures generated by having peptides employing stronger cohesive forces may partially explain the difficulties encountered using techniques that typically work with the phospholipid vesicles. These structures, while sharing certain properties with their lipid counterparts, appear different with regard to initial size, physical stability with regard to the temperature (DSC) or the addition of proteases, chaotropes and dilution.

The rapid cellular uptake of the vesicles and accumulation within the perinuclear space indicates that these structures are capable of delivering membrane impermeant molecules to the interior of cells. We expect that since they are similar in size to viral particles, larger molecules such as hydrophilic drugs, peptides, proteins and large plasmids could be encapsulated and taken up by cells. Peptide nanoparticles, and more importantly peptide nanovesicles, are potential drug delivery systems that can be designed to achieve greater stability and specificity when compared to traditional delivery protocols. The peptides described here are relatively short, stable and easy to prepare. The ability of both short and longer peptides to associate, hints at possibilities of controlling the overall size of the assembly, which will be a useful property when delivering compounds of different sizes. Whereas lipid-based vesicles require additional steps, such as extrusion through polycarbonate filters with defined pore sizes or sonication to achieve a homogeneous distribution, the peptide assemblies described here do not require such treatments.

## Materials and Methods

### Materials

Dichloromethane, Dimethylformamide, Piperidine, n-Methylpyrrolidone, Trifluoroacetic acid and Acetonitrile were purchased from American Bioanalytical (Natick, MA). Diethyl ether, and 2,2,2-Trifluoroethanol, were purchased from Fisher Scientific (Fairlawn, NJ), all Fmoc protected amino acids were purchased from Anapsec, Inc. (Fremont, CA), CLEAR-amide resin was purchased from Applied Biosystems (Foster City, CA), 5(6)-Carboxyfluorescein was purchased from Fluka (Sigma-Aldrich, St. Louis, MO), 200 proof ethanol was purchased from Decon Laboratories, Inc.(King of Prussia, PA), Amicon ultra-0.5 mL, 30 K MWCO Centrifugal units with regenerated cellulose filters were purchased from Millipore (Billerica, MA).

### Peptide Synthesis

All peptides were generated by solid-phase synthesis with 9-Fluorenylmethoxycarbinyl chemistry as described previously (32). The completed peptides, were cleaved with simultaneous side-chain deprotection by treatment with water in 95% trifluoroacetic acid for 2 h at room temperature. The cleaved peptides are then washed four times with diethyl ether and dissolved in water, and then lyophilized. The peptides were purified by reversed phase HPLC and characterized by matrix-assisted laser desorption time of flight mass spectroscopy (MALDI TOF/TOF). All lyophilized peptides were stored at RT until time of use. For the mercury containing peptides, an additional cysteine was incorporated at the C-termini of both the h_5_ and h_9_ peptides. Following cleavage and deprotection, the cysteine thiol of the two peptides were reacted with 1 eq of methylmercury iodide for 6 h at pH 9.8 as previously described (Gruen, 1970) [39]. Since the product was going to be diluted with unlabeled peptides, the product was used without further purification.

### Molecular Modeling

Molecular dynamics (MD) simulations of peptide and DPPC bilayers were carried out in CHARMM [40], [41] using the MARTINI coarse-grained (CG) force field [42], [43]. The model bilayers contain 128 peptide or DPPC molecules, solvated in about 5000 CG waters. The equilibrium properties of the bilayers were calculated from 100 ns MD simulations at 320 K. Additional 100-ns MD simulations were performed at elevated temperatures from 350 K up to 1000 K (every 50 K) to investigate the thermo-stability of these model bilayers. Peptide or lipid diffusion constants and order parameters were monitored to infer bilayer integrity. The self-assembly simulations were initiated from homogeneously dispersed systems that contain about 10000 CG waters and 128 branched peptide or DPPC molecules. The simulations were carried out at 500 K until self-assembly occurred, which took up to 200 ns for both sequences. The final state often contains small aggregates besides the bilayer. While a single neat bilayer is expected for the most stable state, these final states are kinetically stable and much longer simulations would be required to observe full incorporation of all peptides into a single bilayer.

### TEM Sample Preparation

For phosphotungstic acid (PTA) staining, 15 µL of the peptide vesicle solution (prepared to a final concentration of 1.6 mM of each peptide) was placed on a copper grid (which in turn was placed on a piece of parafilm in a petri dish) and allowed to stand for 3 min. Excess peptide solution was wicked off using filter paper, followed by the addition of 15 µL of 10% PTA to the grid, and allowed to stand for 5 min. Excess PTA was wicked off using filter paper, and the grid was washed lightly with DDI water using a syringe. Excess water was wicked off and the grid was allowed to dry before imaging.

For osmium tetraoxide staining, 7 µL of the peptide vesicle solution (final concentration of 25 µM of each peptide) was placed on a copper grid (which in turn was placed on a piece of parafilm in a petri dish) and allowed to stand for 10 min. A drop of osmium tetroxide was placed on the parafilm, a few inches away from the copper grid containing the peptide sample. The petri dish was covered to allow the OsO_4_ vapors to deposit on the peptide sample for 5 min. These grids were then allowed to dry before imaging.

### STEM Sample Preparation

For STEM sample preparation the vesicles were prepared with 30% CH_3_-Hg label in both the h5 and h9 peptides at 0.1 mM concentration were negatively stained using a multi isotope 2% Uranyl acetate (Uranium bis(acetato)-O)-dioxodihydrate) aqueous solution. This was placed on a sample holder and wicked to removed excess solvent and then allowed to air dry. The FEI Tecnai F20 XT Field Emission Transmission Electron Microscope with 0.18 nm STEM HAADF resolution and a magnification range of 150 X –230×10^6^ X was used to perform Scanning Transmission Electron Microscopy at a 17° tilt. The images were captured using annular dark field mode was then inverted to produce the final image.

### Dynamic Light Scattering

h_9_h_5_-vesicles were prepared at a 1 mM concentration. The rehydrated samples were allowed to stand at room temperature for 2 h before filtering through a 0.22 µ filter to remove any dust particles that might be present. 5 mL of the sample was filtered directly into a clean glass tube. The custom built light scattering setup consisted of an argon ion laser operating at 488 nm. The vertically polarized incident beam was focused onto the sample using a lens while the scattered light was collected using a second lens and imaged onto a 300 µm pinhole. All experiments were performed at room temperature. The scattered light was collected at four different angles; the data shown here is that for 30°C. The diffracted light from the pinhole was collected using a photomultiplier tube. A commercial correlator was used to calculate the scattered-light intensity autocorrelation function. The hydrodynamic radius of the sample was obtained by performing curve fitting and analyzing the distribution, using Dynals (performed by Dr. Louis R. Nemzer) yielded a mean auto correlation time of 1.85 ms for the major distribution. This autocorrelation time was used to calculate the hydrodynamic radius from the Stokes-Einstein equation.
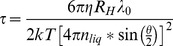
where, **τ**: Correlation time; **η**: Viscocity (0.7978×10^−3^ P.s); **R_H_**: hydrodynamic radius (m); λ_0_ =  wavelength (cm^−1^); **k**: Boltzman constant (1.38×10^−23 ^J K^−1^); **T**: Temperature (300°K); **n_liq_** is the refractive index of the solution (1.33); θ  =  the angle at which the detector was located with respect to the sample (30°C).

### Circular Dichroism

All spectra were collected in a Jasco J-815 CD spectrophotometer using a circular quartz cuvette with a pathlength of 1.0 mm from 260 nm to 190 nm at room temperature. The spectra were an average of five scans recorded at a scan rate of 50 nm/min with a 1 nm step interval and measured in mdeg. Raw scans were processed (subtracted from blank and smoothed) using Spectra Analysis (registered software from Jasco). All peptide concentrations were determined measuring the phenylalanine absorbance of stock solutions at 257.5 nm, then diluting them to the desired final concentration.

### ATR-FTIR

Was performed on a lyophilized sample of the h_9_h_5_-vesicles. A Nicolet 380 (Thermo Scientific, FL), ATR-FTIR fitted with a ZnSe crystal was used to collect the spectra of the sample. Spectra was collected at a resolution of 1 cm^−1^ and averaged 64 times. The collected spectra were plotted using SigmaPlot.

### Differential Scanning Calorimetry

DSC experiments were performed with a VP-DSC calorimeter (MicroCal Inc., Northampton, Massachusetts). Thermograms were obtained at the 1 K/min scan rate for the peptide samples at 1.0 mg/mL. The instrument baseline was obtained first by measuring the thermogram of water. Subsequently, water in the sample cell was replaced with a peptide solution and the sample thermograms were measured.

### 5(6)-Carboxyfluorescein Encapsulation and Release

Two sets of h_9_h_5_-vesicle samples were prepared where, individual peptides dissolved in 40% TFE were mixed in an equimolar ratio to a final concentration of 50 µM and dried under vacuum. One set of dried h_9_h_5_-vesicle samples were dissolved in increasing amounts of 5(6)-Carboxyfluorescein (0.1 mM –2.5 mM) in pure ethanol (20 µL). After allowing these samples to sit at RT for 1 h, they were diluted with drop wise addition of water until the final ethanol concentration was 10% and a final volume of 200 µL. The second set, where the peptide vesicles are allowed to be formed before the addition of 5(6)-Carboxyfluorescein were initially dissolved in pure ethanol (10 µL) and diluted by the drop wise addition of water (180 µL). 5(6)-Carboxyfluorescein dissolved in pure ethanol (10 µL) was added at the final step. Controls with 5(6)-Carboxyfluorescein without any peptide vesicles were prepared in parallel.

Samples for studies involving encapsulation of 5(6)-Carboxyfluorescein in different ethanol concentrations were prepared in a similar manner as described above. Briefly, dried h_9_h_5_-vesicle samples were dissolved in 5(6)-Carboxyfluorescein (0.5 mM) in pure ethanol (10 µL) and diluted to the appropriate final ethanol concentration (10%–70%) by the drop wise addition of water and a final volume of 200 µL.

All samples were allowed to stand at RT for 2 h before centrifuging them through a 30-kDa molecular weight cut off (MWCO) filter at 14,000 g for 10 min. After the MWCO separation, the retentate and filtrate were dried under vacuum and brought up in a solution of pure TFE. These samples were immediately sealed with parafilm, vortexed and allowed to sit at room temperature for 2 h before their fluorescence emission scans were collected by exciting the sample at 493 nm (for 5(6)-Carboxyfluorescein) and 259 nm (for the peptide) in a CARY Eclipse fluorescence spectrophotometer (Varian Inc., Palo Alto, CA).

### Cell Culture and Dye Uptake

N/N 1003A rabbit lens epithelial cells were obtained from Dr. Dolores Takemoto (Kansas State University). These adherent cells were grown to roughly 80% confluence on coverslips placed in twelve well plates with 1 mL Dulbecco’s Modified Eagle Medium (DMEM). Stock solutions of bis(h_9_)-K-K_4_ and bis(h_5_)-K-K_4_ dissolved in DDI water were mixed together using the protocol described above to achieve a final concentration of 0.05 mM of each peptide in the mixture and 1000 µL final volume. The sample was then dried in a speed vacuum apparatus for about 3 h until almost all of the solvent was removed. For the fluorescent dye uptake experiments, the dried sample was then rehydrated with 100 µL of 0.5 mM solution of the fluorescent dye 5(6)-Carboxyfluorescein (MW = 376.3 Da) dissolved in DDI water. The pH of the sample was adjusted to 7.4 using a dilute solution of NaOH. Next, 500 µL of the DMEM was replaced with the peptide vesicle solution loaded with 5(6)-Carboxyfluorescein. The cells were then allowed to grow at 37°C under 5% CO_2_. At 10 h after incubation with dye-loaded vesicles, the cells were washed twice with phosphate buffered saline (PBS) and fixed using 3.7% paraformaldehyde. The fixed cells were then mounted on a glass slide and pictures were taken using a fluorescence microscope. Control cells were incubated with only the free fluorescent dye and no peptide vesicles, followed by washing twice with PBS and fixed with paraformaldehyde.
